# Spike in Congenital Syphilis, Mississippi, USA, 2016–2022

**DOI:** 10.3201/eid2910.230421

**Published:** 2023-10

**Authors:** Manuela Staneva, Charlotte V. Hobbs, Thomas Dobbs

**Affiliations:** Mississippi State Department of Health, Jackson, Mississippi, USA (M. Staneva);; University of Mississippi Medical Center, Jackson (C.V. Hobbs, T. Dobbs)

**Keywords:** congenital syphilis, bacteria, neonatal illnesses, prematurity, health disparities, Mississippi, United States, Treponema pallidum

## Abstract

In Mississippi, USA, infant hospitalization with congenital syphilis (CS) spiked by 1,000%, from 10 in 2016 to 110 in 2022. To determine the causes of this alarming development, we analyzed Mississippi hospital discharge data to evaluate trends, demographics, outcomes, and risk factors for infants diagnosed with CS hospitalized during 2016–2022. Of the 367 infants hospitalized with a CS diagnosis, 97.6% were newborn, 92.6% were covered by Medicaid, 71.1% were African American, and 58.0% were nonurban residents. Newborns with CS had higher odds of being affected by maternal illicit drug use, being born prematurely (<37 weeks), and having very low birthweight (<1,500 g) than those without CS. Mean length of hospital stay (14.5 days vs. 3.8 days) and mean charges ($56,802 vs. $13,945) were also higher for infants with CS than for those without. To address escalation of CS, Mississippi should invest in comprehensive prenatal care and early treatment of vulnerable populations.

Congenital syphilis (CS), caused by infection with the bacterium *Treponema pallidum*, is a severe disease with potential for immediate and long-term health complications. Infection in pregnant mothers can lead to serious neonatal conditions, such as deformities, hepatosplenomegaly, anemia, jaundice, and failure to thrive (*1*). Even though syphilis can be asymptomatic in infants at birth, later sequalae, such as neurologic disorders, occur in ≈40% of untreated children (*2*). In addition, syphilis has been associated with severe pregnancy outcomes, including spontaneous abortion, preterm delivery, stillbirth, and infant death (*3*).

According to Centers for Disease Control and Prevention surveillance data, the nationwide rate of CS increased by 30.5% in 1 year, from 59.7/100,000 live births in 2020 to 77.9/100,000 live births in 2021 (*4*). In some states, CS rates increased even more dramatically. In Mississippi, for example, CS incidence rose from 104.3/100,000 live births in 2020 to 182.0/100,000 live births in 2021, a 74.5% jump in a single year. For this study, we examined trends, demographics, risk factors, coexisting conditions, and outcomes among infants in Mississippi hospitalized with a CS diagnosis. We aimed to better understand this emerging public health crisis in a state that continues to experience deep social and health inequities. 

## Methods 

### Data

For this retrospective cross-sectional study, we analyzed hospital discharge data from Mississippi for 2016–2022. The Mississippi State Department of Health, in collaboration with the Mississippi Hospital Association, collects those data and shares them with the Agency for Healthcare Research and Quality Healthcare Cost and Utilization Project as part of the organization’s national hospital care database (*5*). The rich population-level data contain demographic, clinical, and financial information derived from billing claims submitted by all nonfederal acute-care hospitals in Mississippi. 

### Case Definition

For this study, we included infants ≤1 year of age living in Mississippi. To meet the goals of our investigation, we created 2 study groups. The first comprised all infants hospitalized with CS, including those diagnosed at delivery hospitalization and those who were admitted after delivery (postdelivery subcohort). We used data from this more inclusive group to study prevalence, trends, and demographics among infants hospitalized with CS. The second group (delivery subcohort) included only infants either delivered in the hospital or delivered outside of the hospital but admitted with a delivery code from the International Classification of Diseases, 10th Revision, Clinical Modification (ICD-10-CM). We needed data from the delivery subcohort because we were interested in pregnancy outcomes associated with syphilis, including preterm and low-birthweight deliveries. To identify the delivery subcohort, we developed an algorithm that incorporated infant age and ICD-10-CM code Z38, the neonatal diagnostic code for birth. In hospital discharge data, neonatal patient age was based on date of admission. In our delivery subcohort, we included all infants whose ages were recorded as 0–3 days with day 0 being the day of birth. 

### Data Variables

We used ICD-10-CM code A50 to identify diagnoses of infant hospitalizations with CS. We referenced related ICD-10-CM codes to study newborns affected by maternal use of illicit substances: opiates (P04.14), amphetamines (P04.16), cocaine (P04.41), hallucinogens (P04.42), cannabis (P04.81), unspecified drugs of addiction (P04.40, P04.49), and neonatal abstinence syndrome (P9.61; i.e., severe withdrawal symptoms). For preterm deliveries, we referenced codes P0.72 for extreme newborn immaturity (<27 completed weeks of gestation) and P0.73 for premature births (<36 completed weeks of gestation). We referenced ICD-10-CM code P22 to screen the data for newborn respiratory distress, which were of special interest for this study because this condition is a major cause of neonatal illness and death (*6*). To evaluate birthweight, we used the variable for birthweight in the dataset. Likewise, we detected deaths of infants with diagnosed CS based on the dataset variable for discharge status, which includes in-hospital deaths. Finally, we used neonatal ICD-10-CM code P95 to identify stillbirths and screened those records for CS diagnoses. 

For our research, we collected key demographic characteristics, such as urbanicity, race, and socioeconomic status, for each individual CS patient. We defined urban versus nonurban status on the basis of the CDC National Centers for Health Statistics urban–rural classification scheme (*7*), in which counties with >50,000 inhabitants are classified urban and counties with <50,000 nonurban (10,000–49,999, micropolitan; <10,000, rural). We categorized data on race into 3 groups: African American, White, and others (because few infants with CS in the study were not either African American or White). The following racial categories are included in the hospital discharge dataset: White, African American/Black, American Indian/Alaska Native, Asian, Native Hawaiian/other Pacific Islander, multiracial, declined, and race unavailable/unknown. We were unable to examine ethnicity because few infants of Hispanic origin were included in the CS cohort. We used Medicaid insurance coverage as a proxy for low socioeconomic status. 

### Statistical Analyses

We used data from individual patients as units of analysis. To select individual patients, we used unique identifying numbers and flagged the first hospitalization for each patient. We included only data from first admissions, not transfers or readmissions. Our analyses included descriptive and inferential statistics. We compared demographic and clinical characteristics between patients with and without CS using independent t-tests for continuous variables and χ^2^ tests for categorical variables. To evaluate the strength of association between different demographic and clinical factors and CS, we performed multivariable regression and obtained adjusted odds ratios (aORs) (*8*). We conducted all analyses using SAS 9.2 statistical software (*9*). 

## Results

During the 2016–2022 study period, 367 infants were hospitalized in Mississippi with diagnosed CS. Of those infants, 97.6% were newborn (<28 days), 92.6% covered by Medicaid, 71.1% African American, and 58.0% residents of nonurban counties; 50.7% were male and 49.3% female. Most infants with CS (340; 92.6%) were identified as delivery hospitalizations and included in the delivery subcohort. The remaining 27 infants were postdelivery admissions. Among the postdelivery admissions, 18 were newborn and 9 were >28 days of age, meaning ≈7% of the infants with CS received the diagnosis during postdelivery hospitalization.

### Trend Analyses

**Figure 1:**
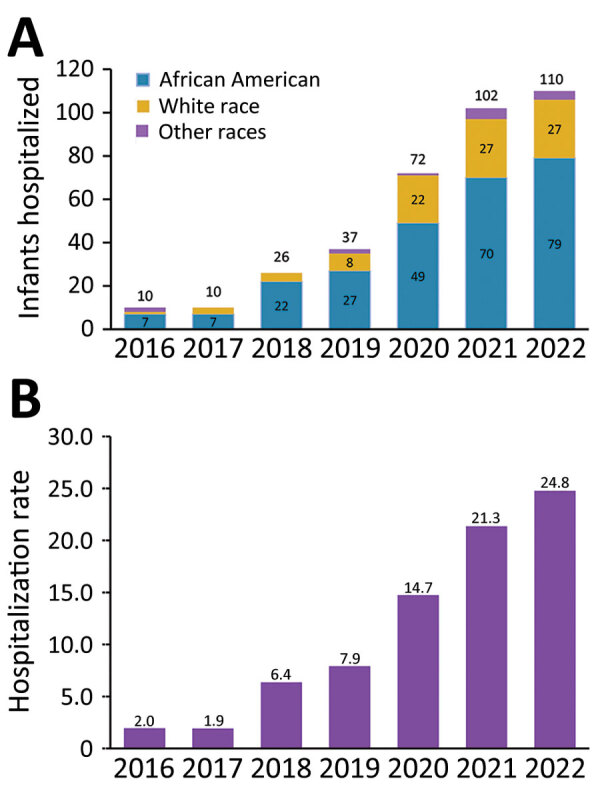
Infants hospitalized with congenital syphilis, Mississippi, USA, 2016–2022. A) Number of infants hospitalized by year and race. B) Hospitalization rates for congenital syphilis per 10,000 delivery hospitalizations. Numbers above bars are rates for each year. We obtained the rates by dividing the number of infants hospitalized with congenital syphilis by the total number of delivery hospitalizations per year.

The number of infants hospitalized with CS spiked by 1,000% over the study period, rising sharply from 10 infants in 2016 to 110 in 2022 (Figure 1, panel A). The rate of hospitalization of infants with CS increased from 2.0/10,000 total infant hospitalizations in 2016 to 24.8/10,000 total infant hospitalizations in 2022, representing a 1,140% increase (Figure 1, panel B). Additional analyses revealed 2 notable trends. First, the rate increase in the number of infants hospitalized with CS varied by race. Although infants with CS were overwhelmingly African American, the magnitude of increase was higher among White infants. Among African American infants, CS hospitalizations increased by 1,029%, rising from 7 infants in 2016 to 79 in 2022. Over the same time period, the admissions among White infants jumped by 2,600%, from 1 infant in 2016 to 27 in 2022. Second, hospitalization for infants with CS started to increase in 2018, before the onset of the COVID-19 crisis; the pandemic, however, accelerated this uptrend. 

### Demographic Risk Factors 

Bivariate analyses of associations between CS and different demographic characteristics revealed that infants with CS were more likely than those without to be African American (71.1% vs. 43.3%; p<0.001), covered by Medicaid (92.6% vs. 63.9%; p<0.001), and >3 days of age (7.4% vs. 2.0%; p<0.001). After adjusting for covariates, Medicaid insurance coverage (aOR, 5.24, 95% CI, 3.58–8.00) was the strongest independent demographic risk factor; however, infants >3 days of age (aOR, 3.82, 95% CI, 2.52–5.55) and African American race (aOR, 2.26, 95% CI, 1.37–4.06) also retained associations with congenital syphilis risk (Table 1).

**Table 1:** Demographic characteristics of infants hospitalized with and without congenital syphilis diagnosis, Mississippi, USA, 2016–2022

Patient characteristics	Congenital syphilis, no. (%)	No congenital syphilis, no. (%)	p value	Crude odds ratio (95% CI)	Adjusted odds ratio (95% CI)
Total	367	238,227			
Age group, d					
0–3	340 (92.6)	233,528 (98.0)	<0.001	Referent	Referent
4–365	27 (7.4)	4,699 (2.0)		3.947 (2.60–5.73)	3.82 (2.52–5.55)
Sex†				Referent	Referent
F	179 (49.0)	116,615 (49.0)	0.981	1.003 (0.82–1.23)	1.01 (0.82–1.24)
M	186 (51.0)	121,483 (51.0)		Referent	Referent
Race					
African American	261 (71.1)	103,102 (43.3)	<0.001	2.83 (1.72–5.07)	2.26 (1.37–4.06)
White	92 (25.1)	119,502 (50.2)		0.86 (0.51–1.58)	1.01 (0.60–1.86)
Other	14 (3.8)	15,623 (6.5)		Referent	Referent
Residence					
Nonurban	213 (58.0)	133,829 (56.2)	0.472	1.08 (0.88–1.33)	1.02 (0.83–1.26)
Urban	154 (42.0)	104,398 (43.8)		Referent	Referent
Primary expected payer					
Medicaid	340 (92.6)	152,221 (63.9)	<0.001	7.11 (4.91–10.78)	5.24 (3.58–8.00)
Private/other	27 (7.4)	86,006 (36.1)		Referent	Referent

### Clinical Characteristics of the Delivery Cohort

We compared findings on clinical characteristics from this analysis between infants with and without CS (Table 2). In the bivariate analysis, newborns with CS were significantly more likely than those without CS (21.5% vs. 2.4%; p<0.001) to have a maternal illicit drug use ICD-10-CM code on record. Among newborns with CS, 73 were affected by maternal substance use; however, only 36/73 (49.3%) records for those newborns included a substance-specific diagnostic code. Among those 36 infants, maternal use of cannabis was recorded for 69.4%, cocaine 27.8%, amphetamines 25.0%, and opioids 2.8%. Drug-use categories were not mutually exclusive, and 7/36 CS-infected newborns with recorded substance-specific diagnostic codes were exposed to >1 substance in utero. Neonatal abstinence syndrome was present in 8 cases. 

**Table 2:** Delivery subcohort: clinical characteristic for infants with and without congenital syphilis, Mississippi, USA, 2016–2022

Infant characteristics	Congenital syphilis, no. (%)	No congenital syphilis, no. (%)	p value	Crude odds ratio (95% CI)	Adjusted odds ratio (95% CI)*
Maternal substance use	340	233,529	<0.001		
Y	73 (21.5)	5,597 (2.4)		11.14 (8.53–14.36)	9.39 (7.16–12.16)
N	267 (78.5)	227,932 (97.6)		Referent	Referent
Birthweight†					
Very low birthweight	28 (8.5)	4,430 (1.9)	<0.001	5.38 (3.55–7.81)	4.05 (2.67–5.90)
Low birthweight	59 (17.9)	22,556 (9.7)		2.23 (1.66–2.94)	1.81 (1.35–2.40)
Normal birthweight	242 (73.6)	205,901 (88.3)		Referent	Referent
Preterm					
Y	92 (27.1)	29,544 (12.7)	<0.001	2.56 (2.01–3.24)	2.26 (1.77–2.86)
N	248 (72.9)	203,985 (87.3)		Referent	Referent
Newborn respiratory distress					
Y	72 (21.2)	21,204 (9.1)	<0.001	2.69 (2.06–3.47)	2.54 (1.94–3.28)
N	268 (78.8)	212,325 (90.9)		Referent	Referent

*Each of the models was adjusted for demographic characteristics, including race, sex, residence, and payer. We excluded newborns of undetermined/unknown sex from this analysis. †Not all newborn records had recorded birthweight; therefore, we excluded newborns with unknown birthweight from this analysis (11 records for the congenital syphilis cohort and 642 records for the cohort without congenital syphilis). Very low birthweight, <1,500 g; low birthweight, 1,500-2,500 g; normal birthweight, >2,500 g.

**Figure 2:**
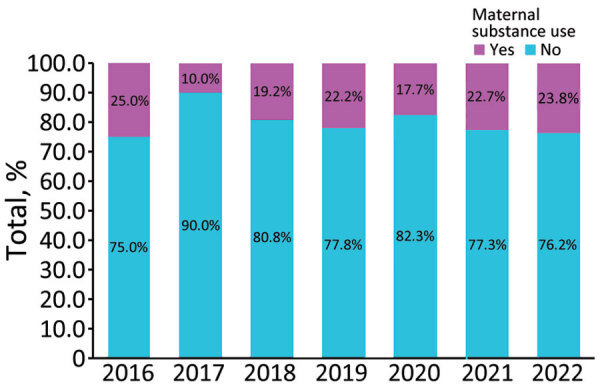
Maternal illicit drug use among newborns with congenital syphilis, Mississippi, USA, 2016–2022.

In the multivariable analysis of clinical characteristics, the variable with the strongest association with CS was maternal substance use. Even after controlling for demographic risk factors, the CS cohort had almost 10 times higher odds of being affected by maternal illicit drug use than the cohort without CS (aOR, 9.39, 95% CI 7.16–12.16). Aside from minor variations, the proportion of maternal illicit drug use among the CS cohort remained high throughout the study period. In 2016, the year with the highest proportion of substance-exposed infants, 25% of all newborns with CS also had a diagnostic code indicating maternal use of illicit drugs. During the last 3 years of the study period, there was a gradual but steady increase in the proportion of newborns hospitalized with CS and exposed to illicit substances in utero: 17.7% in 2020, 22.7% in 2021, and 23.8% in 2022 (Figure 2). 

**Figure 3:**
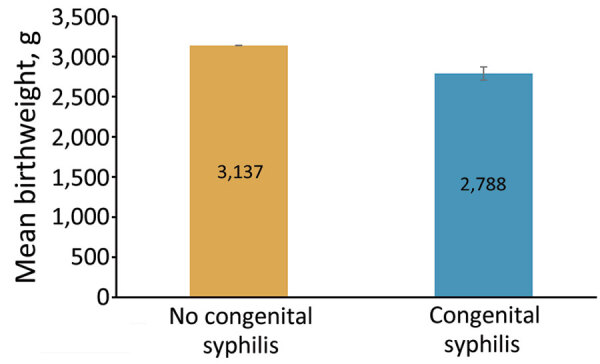
Mean birthweight for newborns with and without congenital syphilis, Mississippi, USA, 2016–2022. Error bars indicate 95% CIs.

Infants in the CS cohort were also more likely to suffer from severe complications, including prematurity and low birthweight, than infants in the cohort without CS. Among newborns with CS, 92 (27.1%) were premature, compared with 43 (12.7%) for newborns without CS. Among premature infants with CS, 8/92 were extremely premature (i.e., born before completing 28 weeks of gestation). Newborns with CS had >2 times higher odds of being born prematurely than did newborns without CS (aOR 2.26, 95% CI 1.77–2.86) after controlling for demographic risk factors. Among 87 newborns with CS who had birthweights <2,500 g, 59 had low birthweight (LBW) of 1,500–2,499 g and 28 had very low birthweight (VLBW) of <1,500 g. Compared with the cohort without CS, infants in the CS cohort were more likely to be LBW (17.9% vs. 9.7%; p<0.001) and VLBW (8.5% vs. 1.9%; p<0.001). Alarmingly, odds for newborns with CS to be LBW were nearly 2 times higher (aOR 1.81, 95% CI 1.35–2.40) and to be VLBW >4 times higher (aOR 4.05, 95% CI 2.67–5.90) than for newborns without CS. On average, newborns with CS weighed 349 g less (mean birthweight 2,788 g) than newborns without CS (3,137 g; p<0.001) (Figure 3). Newborn respiratory distress was also more common among the CS cohort (21.2%) than for the cohort without CS (9.1%; p<0.001). Given the well-established connection between prematurity and respiratory distress in newborns, it is unsurprising that 1 in 5 premature newborns with CS in our study was also diagnosed with respiratory distress. Among the 72 newborns with diagnoses for CS and respiratory distress, 48 (66.7%) also had a diagnostic code for preterm delivery. 

### Use of Resources 

In terms of resource use, infants with CS had a longer mean hospital stay (14.5 days vs. 3.8 days; p<0.001) and mean hospital charges ($56,802 vs. $13,945; p<0.001) than did infants without CS. During 2016–2022, hospital charges for the CS cohort totaled $20,846,196; Medicaid-insured infants accounted for $19,444,608 (93.3%) of those charges.

### Mortality

During the last 4 years of the study period (2019–2022), 6 infants with CS died in hospital, half of those during the last year of the study period (2022). Among the infants with CS who died, 83.3% had extremely low birthweight (<1,500 g), and 66.7% were extremely premature. Among our study population, no records indicated both stillbirth and CS. 

## Discussion

From 2016 through 2022, CS hospitalization rates in Mississippi increased more than 10-fold. Although the upward trend was consistent with national-level surveillance data for CS, the upturn in Mississippi was even steeper (*10*). This spike is troubling because decades of research have demonstrated the dire health consequences of CS, including prematurity, low birthweight, and death (*11*).

As CS rates in Mississippi rose steeply in recent years, associated harmful health outcomes contributed to the already high state infant illness and death burden. In our study, the percentage of premature infants among the CS cohort (27.1%) was >2 times higher than for the cohort without CS (12.7%). Likewise, the CS cohort had a higher proportion of newborns with VLBW. We acknowledge that other associated risk factors more commonly seen in the CS cohort, such as maternal substance abuse, might also have contributed to this difference. Still, those differences require urgent action because preterm birth and VLBW are leading causes of infant death in Mississippi, a state that already has the highest preterm birth and infant mortality rates in the nation (*12*). 

Our study provides evidence that the spike in CS is already elevating the high number of infant deaths in the state. Our research identified 6 infant deaths associated with a CS diagnosis during the study period. Half those deaths occurred in 2022, underlying the current nature of this rapidly escalating reemergent public health menace and urgent need for public health interventions. Infant deaths associated with CS are preventable; Mississippi could reduce infant illness and death by promptly diagnosing and treating pregnant patients with syphilis (*13*). 

In addition to harmful health sequalae, CS was associated with significant healthcare expenditure as measured by the mean length of stay and hospital charges. The mean length of stay was nearly 4 times higher among the CS cohort than for the cohort without CS. Furthermore, total charges for this preventable condition reached >$20 million during the 7-year study period. This result further underlines the pressing need to prevent CS to reduce its physical and economic toll in Mississippi. 

To combat this emerging crisis, however, it is critical to understand the different behavioral, social, and economic factors driving up maternal syphilis rates (*14*–*16*). In Mississippi, for example, the rise in the CS rate paralleled another concerning trend: rapid increase in newborn hospitalization associated with maternal substance use (*17*). In our study, nearly 1 in 4 infants with CS was born to a mother with a substance use disorder. This finding reveals the entanglement between the ongoing drug epidemic and the resurgence of maternal and congenital syphilis and suggests the need for holistic approaches that treat illicit drug use as one way to curtail CS rates (*18*).

We further uncovered substantial economic and racial disparities among families of infants diagnosed with CS, consistent with previous research (*19*). In our study, >70% of infants hospitalized with CS were African American, and nearly all (91%) infants with this diagnosis were insured by Medicaid. Our demographic analysis demonstrates the intertwined nature of social, economic, and racial disparities with health outcomes and highlights the roles of poverty and institutional racism in fueling transmission of severe but preventable infections, such as syphilis (*20*). 

In addition to behavioral and socioeconomic risk factors, insufficient prenatal screening has been implicated in rapidly rising CS rates (*21*,*22*). Until March 2023, Mississippi was one of only a few states not mandating syphilis screening for prospective mothers (*23*,*24*). By contributing to underscreening, this policy omission led to missed opportunities to detect and treat maternal syphilis before the birth of infants. Despite being a resource-constrained state with a history of profound health disparities, Mississippi has neither expanded Medicaid nor adopted presumptive eligibility for uninsured pregnant persons. Failure to pursue such policies created more structural barriers for early and effective prenatal care that enabled the syphilis problem to persist and grow. Redirected public health efforts and disrupted medical care because of the COVID-19 pandemic also played roles in ratcheting up CS incidence by impeding prevention efforts and consequentially precipitating increased syphilis transmission (*25*,*26*). Those and other obstacles to timely prenatal testing and care have delayed diagnosis and treatment of syphilis case-patients during pregnancy. 

To address the evolving CS crisis in Mississippi effectively, comprehensive public health and health policy approaches must be implemented. State agencies and political leaders should be encouraged to adopt all available tools to support early comprehensive prenatal care. At the front line of this battle, public health structures should receive adequate resources to diagnose and treat the most vulnerable populations, including underserved and uninsured/underinsured infected mothers, in a timely and effective manner. In addition, healthcare providers and systems should align their processes to prioritize and ensure early diagnosis and treatment of syphilis in pregnancy. Enhancing sexual health literacy, including providing comprehensive sex education for youth, would further empower Mississippi residents to prevent or seek treatment for reemerging syphilis infection. 

Our investigation shows that the rapid rise of CS hospitalizations in Mississippi is a serious public health concern because of its grave social and health-related consequences. By analyzing hospital discharge records from Mississippi, we performed an in-depth epidemiologic evaluation of infants hospitalized with CS. Although limited by lack of data on prenatal care, perinatal syphilis treatment, and laboratory diagnostic tests, the database provided population-level data that enabled us to explore statewide trends, study comorbidities and costs, and make robust inferences specific to the population of Mississippi. Study findings suggest the benefits of obtaining more granular data, improving public health surveillance, and enhancing education for physicians and providers to diagnose and treat syphilis. 

Congenital syphilis is a disease with dire health, social, and financial consequences that can be prevented. Providing comprehensive prenatal care, effective screening, and early treatment for pregnant women in Mississippi constitute not only sound public health policy in general but also smart strategies to improve pregnancy outcomes, reduce infant illness and death, curtail medical costs, and promote greater health equity. 
